# Prognostic significance of preoperative gamma-glutamyltransferase to lymphocyte ratio index in nonfunctional pancreatic neuroendocrine tumors after curative resection

**DOI:** 10.1038/s41598-017-13847-6

**Published:** 2017-10-17

**Authors:** Bo Zhou, Canyang Zhan, Jingjing Wu, Jianhua Liu, Jie Zhou, Shusen Zheng

**Affiliations:** 1 Division of Hepatobiliary and Pancreatic Surgery, Department of Surgery, First Affiliated Hospital, School of Medicine, Zhejiang University, Hangzhou, China; 2 Department of Neonatology, Children’s Hospital, School of Medicine, Zhejiang University, Hangzhou, China; 3 Department of Pathology, First Affiliated Hospital, School of Medicine, Zhejiang University, Hangzhou, China

## Abstract

Various inflammation-based prognostic scores have been associated with reduced survival in patients with nonfunctional pancreatic neuroendocrine tumor (NF-PNET). However, few studies have illuminated the relationship between the preoperative gamma-glutamyltransferase (GGT) to lymphocyte ratio index (GLRI) and the prognosis of NF-PNET. A retrospective review of 125 NF-PNET patients following curative resection was conducted. The cut-off values for the inflammation-based prognostic scores, including GLRI, were selected using receiver operating characteristic curve analysis. Univariate, multivariate and Kaplan-Meier analyses were used to calculate overall survival (OS) and disease-free survival (DFS). The optimal cut-off value for GLRI was 10.3. Multivariate analysis showed that GLRI was an independent predictor of OS (P = 0.001) and DFS (P = 0.007) for NF-PNET. Kaplan-Meier analysis also showed that preoperative GLRI had significant prognostic value in various subgroups of patients with NF-PNET. The discriminatory capability of GLRI was superior to that of other inflammation-based scores in OS prediction. Furthermore, the predictive range was expanded by incorporating GLRI into the conventional stratification systems, including AJCC staging and WHO classification. These results indicated that preoperative GLRI was an independent predictor for NF-PNET patients undergoing curative resection. The incorporation of GLRI into the existing conventional stratification systems resulted in improved predictive accuracy.

## Introduction

Pancreatic neuroendocrine tumors (PNETs), also known as islet cell tumors, are rare neoplasms that originate in the endocrine tissues of the pancreas^1,2^. PNETs account for approximately 1–2% of all pancreatic neoplasms and 7.0% of all neuroendocrine tumors^3^. PNETs can be classified as either functional or nonfunctional, while nonfunctional PNETs (NF-PNETs) account for 60% to 90% of all PNETs. Unlike functional PNETs with the typical clinical manifestations of hormone overproduction, NF-PNETs often grow to an advanced stage with a large mass, local invasion and distant metastasis, because of nonspecific symptoms in the early stages, such as abdominal pain and distension, nausea and vomiting, abdominal mass, and others^4^. Complete surgical resection of an NF-PNET has been suggested to be the only potentially curative treatment for the disease, similar to pancreatic ductal adenocarcinoma (PDAC). Recently, several studies have found that a large number of host-related factors affect survival in NF-PNETs. Intrinsic tumor characteristics, such as tumor size, stage and grade, Ki-67 index, and lymph node (LN) involvement, have been shown to be associated with clinical outcomes^5–7^. However, most of these factors can only be determined after surgery. Therefore, it is necessary to search for potential prognostic indicators that are available before surgery.

Gamma-glutamyltransferase (GGT) is a membrane bound enzyme essential to glutathione (GSH) metabolism that protects cells from reactive oxygen species^8^. GGT expression is found predominantly on the luminal surface of secretory epithelial cells, especially of the hepato-biliary tract, pancreas and kidneys^9^. Increasing evidence has suggested that an elevated serum GGT level is an independent predictor of poor survival in several cancer types, such as pancreatic cancer^10^, intrahepatic cholangiocarcinoma^11^ and hepatocellular carcinoma^12^. Engelken FJ *et al*. reported that elevated GGT and leukocytosis predicted shorter survival for patients with unresectable pancreatic cancer^10^. Research from Diergaarde B suggested that a common variation in the GGT1 gene could affect the risk of pancreatic cancer^13^. Furthermore, recent data suggested that higher serum levels of GGT, within the normal range, was an early marker of oxidative stress and an indicator of higher cancer risk^14^.

Lymphocyte count, which can comprehensively reflect the systemic inflammatory response in patients with cancer, has been a reliable marker for predicting the survival of patients with different types of cancer^15,16^. Katz SC *et al*. demonstrated that lymphocyte infiltration was common in the majority of NETs, as assessed by immunohistochemistry for CD3, CD4, CD8, and CD56^17^. Tumor-infiltrating lymphocytes (TILs) have been shown to predict outcomes in numerous primary human malignancies, including pancreatic adenocarcinoma, hepatocellular carcinoma and colorectal cancer^18^. In contrast, lymphocytopenia has been reported in various cancers, but is particularly marked in patients with pancreatic cancer. Preoperative lymphocytopenia is a poor prognostic factor in patients with pancreatic cancer^15^. It has been suggested that lymphocytopenia, indicating a state of depressed immune function, may influence survival adversely due to reduced host response to the tumor cells.

In combination with the effects of GGT and lymphocytes mentioned above, the GGT to lymphocyte ratio index (GLRI) may be a potentially effective biomarker for tumor prognosis. Additionally, the GGT to platelet ratio index (GPRI), a predictor of liver fibrosis and cirrhosis^19^, was an independent predictive factor for HBV-related hepatocellular carcinoma after hepatic resection^20^. To our knowledge, the GLRI has not been used to predict the survival and tumor recurrence after curative resection for NF-PNETs. The goal of this study was to assess the prognostic value of GLRI in patients with NF-PNETs following curative resection. Further, we aimed to compare the discriminative ability of GLRI with that of other inflammation scores to determine whether the GLRI could be a useful marker for predicting patients’ outcomes. Additionally, we attempted to refine the existing stratification systems by incorporating GLRI into the existing TNM staging system or WHO classification.

## Results

### Patients’ clinicopathological characteristics

The clinicopathological characteristics are provided in Table 1. This study included 64 male patients (51.2%) and 61 female patients (48.8%). These patients were diagnosed at a mean age of 53.0 ± 12.73 years and were evaluated over a mean follow-up period of 45.76 ± 37.01 months. In this study, 54 patients were identified incidentally during health examinations. The most common presentation of symptomatic PNETs was abdominal pain in 56 (44.8%) patients, followed by abdominal discomfort in 8 (6.4%), obstructive jaundice in 5 (4%) and diarrhea in 2 (1.6%). Only two patients had hepatitis B and were receiving antiviral therapy with entecavir. According to the inclusion criteria, no patients suffered from the cholangitis. The numbers of patients classified into AJCC stages I, II, III and IV were 88, 18, 4 and 15, respectively. The numbers of patients classified into grades 1, 2 and 3 were 41, 62 and 22, respectively. The 1-, 3-, and 5-year OS and DFS rates were 98%, 89%, and 76% and 79%, 66%, and 63%, respectively.

**Table 1:** Relationships between GLRI and clinicopathological characteristics in NF-PNET.

Variables	Cases	GLRI ≤ 10.3	GLRI > 10.3	Univariate analysis		Multivariate analysis
	(N = 125)	(N = 41)	(N = 84)	Χ^2^	P	P
Age (years)				0.106	0.745	
≤60	86	29 (33.7%)	57 (66.3%)			
>60	39	12 (30.8%)	27 (69.2%)			
Gender				17.551	**<0.001**	**<0.001**
Female	61	31 (50.8%)	30 (49.2%)			
Male	64	10 (15.6%)	54 (84.4%)			
Tumor size (cm)				1.195	0.274	
≤2	32	13 (40.6%)	19 (59.4%)			
>2	93	28 (30.1%)	65 (69.9%)			
Tumor location				0.245	0.62	
Head/uncinate/neck	54	19 (35.2%)	35 (64.8%)			
Body/tail	71	22 (31.0%)	49 (69.0%)			
Symptoms				7.857	**0.005**	0.067
Absent	54	25 (46.3%)	29 (53.7%)			
Present	71	16 (22.5%)	55 (77.5%)			
Albumin (g/l)				0.34	0.56	
**<**35	7	3 (42.9%)	4 (57.1%)			
≥35	118	38 (32.2%)	80 (67.8%)			
AKT (U/l)				8.698	**0.003**	**0.003**
≤90.5	92	37 (40.2%)	55 (59.8%)			
>90.5	33	4 (12.1%)	29 (87.9%)			
T-stage				4.709	**0.03**	0.698
T1–2	111	40 (36.0%)	71 (64.0%)			
T3–4	14	1 (7.1%)	13 (92.9%)			
LN metastasis				0.738	0.39	
Absent	98	34 (34.7%)	64 (65.3%)			
Present	29	7 (24.1%)	20 (75.9%)			
Distant metastasis				5.281	**0.022**	0.829
Absent	110	40 (34.8%)	70 (65.2%)			
Present	15	1 (6.7%)	14 (93.3%)			
Perineural invasion				3.950	**0.047**	0.266
Absent	108	39 (36.1%)	69 (63.9%)			
Present	17	2 (11.8%)	15 (88.2%)			
WHO classification				1.072	0.3	
Grade 1	41	16 (39.0%)	25 (61.0%)			
Grade 2–3	84	25 (29.8%)	59 (70.2%)			
AJCC stage				7.708	**0.005**	**0.028**
I-II	106	40 (37.7%)	66 (62.3%)			
III-IV	19	1 (5.3%)	18 (94.7%)			

NF-PNET, nonfunctional pancreatic neuroendocrine tumor; GLRI, gamma-glutamyltransferase to lymphocyte ratio index; AKT: alkaline phosphatase; LN, Lymph node; AJCC, American Joint Committee on Cancer. P-value < 0.05, marked in bold font, shows statistical significance.

### Determination of the cut-off value

Using 5-year overall survival rate as an endpoint, the stratification of GLRI, GPRI, GGT to neutrophil ratio index (GNRI), neutrophil to lymphocyte ratio (NLR), platelet to lymphocyte ratio (PLR) and prognostic nutritional index (PNI) was calculated using receiver operating characteristic (ROC) curve analyses. The results showed the areas under the curve (AUC) for GLRI, GPRI, GNRI, NLR, PLR, and PNI was 0.727, 0.680, 0.622, 0.687, 0.677, and 0.596, respectively, and the optimal cut-off value was 10.3, 28.1, 0.15, 2.6, 140.88, and 46.3, corresponding to the maximum joint sensitivity and specificity, respectively.

### Relationships between GLRI and clinicopathological characteristics

The relationships between preoperative GLRI and the clinicopathological variables of patients with NF-PNET were investigated, and the data showed that preoperative GLRI was correlated with gender (P < 0.001), presence of symptoms (P = 0.005), alkaline phosphatase (AKT) (P = 0.003), T-stage (P = 0.03), distant metastasis (P = 0.022), perineural invasion (P = 0.047), and AJCC stage (P = 0.005). Furthermore, there were no significanct differences between preoperative GLRI and other clinicopathological parameters, such as age, albumin, tumor size, tumor location, LN metastasis and WHO classification (all P > 0.05, Table 1). Multivariate analysis revealed that the independent parameters associated with an elevated GLRI were gender (P < 0.001), AKT (P = 0.003) and AJCC stage (P = 0.028), which suggested that male gender, high AKT, and advanced AJCC stage were associated with elevated GLRI.

### Prognostic significance of GLRI

**Figure 1:**
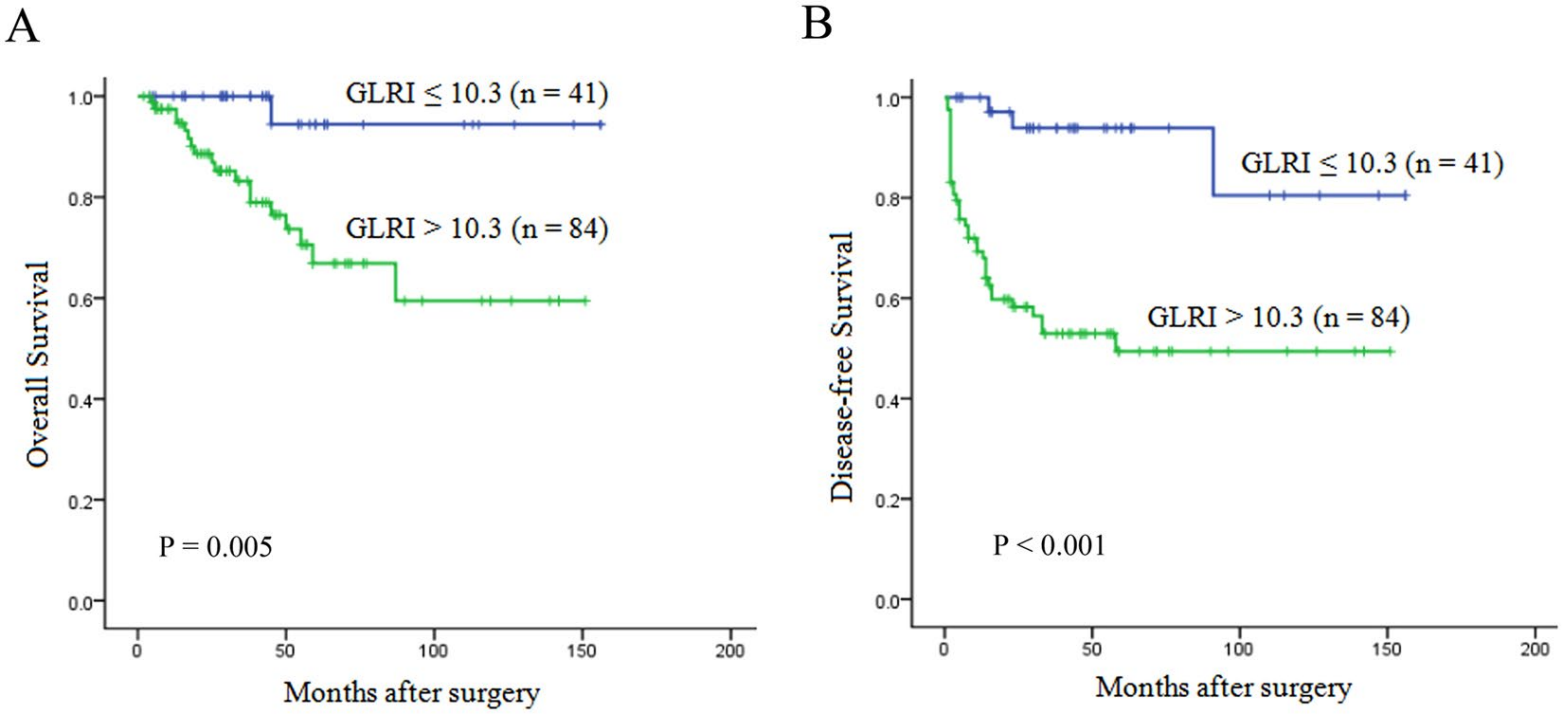
Kaplan-Meier survival curves showing OS (**A**) and DFS (**B**) stratified by GLRI in NF-PNET patients undergoing curative resection. GLRI > 10.3 was significantly correlated with shorter OS and DFS in NF-PNET patients undergoing curative resection.

To determine the ability of preoperative GLRI to predict OS and DFS, the 125 patients were divided into two groups: a low-risk group (GLRI ≤ 10.3, n = 41) and a high-risk group (GLRI > 10.3, n = 84). Using the Kaplan-Meier method to analyse patient survival, the data showed that the 1-, 3- and 5-year OS rates of the low-risk group were markedly higher than those of the high-risk group (100%, 100% and 94.4% vs 94.7%, 83.2% and 66.9%, respectively, P = 0.005) (Fig. 1A), while the 1-, 3- and 5-year DFS rates of the low-risk group were also significantly higher than those of the high-risk group (100%, 93.9% and 93.9% vs 69.3%, 52.9% and 49.4%, respectively, P < 0.001) (Fig. 1B). To further confirm the reliability of GLRI, we analysed the data on postoperative GLRI (7 days after surgery). Most patients with preoperative GLRI > 10.3 still had high postoperative GLRI > 10.3 (n = 75). The Kaplan-Meier analysis showed that patients with high postoperative GLRI had shorter OS (HR = 6.519, 95%CI 1.505–28.247, P = 0.012) and DFS (HR = 5.964, 95%CI 2.332–15.256, P < 0.001) than patients with low postoperative GLRI. Therefore, our research showed that GLRI > 10.3 was correlated with poor survival in patients with NF-PNET undergoing curative resection.

The results of the univariate survival analysis for each of the clinicopathological variables are shown in Table 2. Preoperative GLRI (P = 0.001), GNRI (P < 0.001), GPRI (P = 0.025), NLR (P < 0.001), PLR (P = 0.036), PNI (P = 0.015), symptoms (P = 0.007), AKT (P = 0.007), T-stage (P < 0.001), LN metastasis (P < 0.001), distant metastasis (P = 0.001), perineural invasion (P < 0.001), WHO classification (P = 0.030) and AJCC stage (P < 0.001) were prognostic factors for OS. Similarly, the significant predictors of DFS were GLRI (P = 0.001), GNRI (P = 0.002), NLR (P < 0.001), PLR (P = 0.006), PNI (P = 0.036), symptoms (P = 0.004), gender (P = 0.018), AKT (P = 0.033), T-stage (P < 0.001), LN metastasis (P < 0.001), distant metastasis (P < 0.001), perineural invasion (P < 0.001), WHO classification (P = 0.001) and AJCC stage (P < 0.001).

**Table 2:** Univariate Cox proportional hazards regression models of prognostic factors associated with OS and DFS.

Variables	OS			DFS		
	HR	95% CI	P	HR	95% CI	P
Age (years)			0.895			0.543
≤60	Reference			Reference		
>60	1.072	0.384–2.991		1.228	0.633–2.385	
Gender			0.314			**0.018**
Female	Reference			Reference		
Male	1.616	0.635–4.116		2.221	1.144–4.311	
Symptoms			**0.007**			**0.004**
Absent	Reference			Reference		
Present	7.397	1.708–32.039		2.850	1.391–5.839	
Tumor size (cm)			0.102			0.052
≤2	Reference			Reference		
>2	5.360	0.715–40.174		2.529	0.990–6.459	
Tumor location			0.117			0.541
Head/uncinate/neck	Reference			Reference		
Body/tail	0.481	0.193–1.200		0.822	0.440–1.538	
Albumin (g/l)			0.809			0.83
≤35	Reference			Reference		
>35	1.283	0.170–9.659		1.169	0.281–4.861	
AKT (U/l)			**0.007**			**0.033**
≤90.5	Reference			Reference		
>90.5	3.464	1.404–8.550		1.993	1.057–3.758	
NLR			**<0.001**			**<0.001**
≤2.6	Reference			Reference		
>2.6	5.211	2.080–13.054		3.512	1.876–6.575	
PLR			**0.036**			**0.006**
≤140.88	Reference			Reference		
>140.88	2.717	1.068–6.913		2.415	1.294–4.507	
GLRI			**0.001**			**0.001**
≤10.3	Reference			Reference		
>10.3	8.939	2.602–30.708		7.672	2.360–24.936	
GNRI			**<0.001**			**0.002**
≤28.1	Reference			Reference		
>28.1	6.415	2.596–15.849		2.950	1.465–5.942	
GPRI			**0.025**			0.125
≤0.15	Reference			Reference		
>0.15	3.214	1.156–8.940		1.628	0.873–3.037	
PNI			**0.015**			**0.036**
≤46.3	Reference			Reference		
>46.3	0.328	0.133–0.808		0.507	0.269–0.956	
T-stage			**<0.001**			**<0.001**
T1–2	Reference			Reference		
T3–4	3.805	2.320–6.240		2.466	1.734–3.507	
LN metastasis			**<0.001**			**<0.001**
Absent	Reference			Reference		
Present	18.284	6.015–55.577		4.613	2.467–8.627	
Distant metastasis			**0.001**			**<0.001**
Absent	Reference			Reference		
Present	6.065	2.069–17.779		13.289	6.511–27.124	
Perineural invasion			**<0.001**			**<0.001**
Absent	Reference			Reference		
Present	5.631	2.153–14.729		4.319	2.172–8.586	
WHO classification			**0.030**			**0.001**
Grade 1	Reference			Reference		
Grade 2-Grade 3	5.061	1.168–21.934		11.436	2.756–47.453	
AJCC stage			**<0.001**			**<0.001**
I-II	Reference			Reference		
III-IV	11.228	4.090–30.827		16.581	8.088–33.993	

OS, overall survival; DFS, disease-free survival; AKT, alkaline phosphatase; NLR, neutrophil to lymphocyte ratio; PLR, platelet to lymphocyte ratio; GLRI, gamma-glutamyltransferase to lymphocyte ratio index; GNRI, gamma-glutamyltransferase to neutrophil ratio index; GPRI, gamma-glutamyltransferase to platelet ratio index; LN, lymph node; AJCC, American Joint Committee on Cancer; PNI, prognostic nutritional index. P-value < 0.05, marked in bold font, shows statistical significance.

After multivariate analysis, we found that GLRI, LN metastasis, distant metastasis and WHO classification were independent predictive factors for OS (all P < 0.05), whereas GLRI, WHO classification and AJCC stage were independent predictive factors for DFS (all P < 0.05) (Table 3).

**Table 3:** Independent prognostic factors for OS and DFS identified by the Cox multivariate proportional hazards regression model.

Variables	OS			DFS		
	HR	95% CI	P	HR	95% CI	P
T-stage			0.334			
T1–2	Reference					
T3–4	NA	NA				
LN metastasis			**0.017**			
Absent	Reference					
Present	2.278	1.160–4.471				
Distant metastasis			**<0.001**			
Absent	Reference					
Present	6.089	2.904–12.763				
WHO classification			**0.012**			**0.008**
Grade 1	Reference			Reference		
Grade 2-Grade 3	6.608	1.525–28.630		7.282	1.694–31.309	
Perineural invasion			0.222			0.829
Absent	Reference			Reference		
Present	NA	NA		NA	NA	
Symptoms			0.14			0.246
Absent	Reference			Reference		
Present	NA	NA		NA	NA	
AKT (U/l)			0.086			0.093
≤90.5	Reference			Reference		
>90.5	NA	NA		NA	NA	
GLRI			**0.001**			**0.007**
≤10.3	Reference			Reference		
>10.3	7.425	2.253–24.473		5.310	1.574–17.915	
Gender						0.682
Female				Reference		
Male				NA	NA	
NLR						0.302
≤2.6				Reference		
>2.6				NA	NA	
AJCC stage						**<0.001**
I-II				Reference		
III-IV				10.048	4.620–21.850	

OS, overall survival; DFS, disease-free survival; LN, lymph node; AKT: alkaline phosphatase; GLRI, gamma-glutamyltransferase to lymphocyte ratio index; NLR, neutrophil to lymphocyte ratio; AJCC, American Joint Committee on Cancer; NA, not available. P-value < 0.05, marked in bold font, shows statistical significance.

### Prognostic values of preoperative GLRI in different NF-PNET subgroups

**Figure 2:**
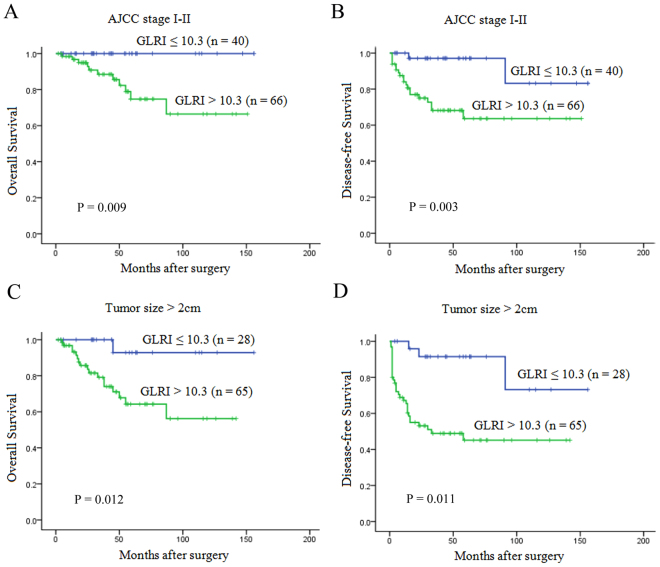
Kaplan-Meier survival curves for the different NF-PNET subgroups. GLRI > 10.3 was significantly correlated with shorter OS and DFS in subgroups with AJCC stage I/II (**A** and **B**) and tumor size > 2 cm (**C** and **D**).

**Figure 3:**
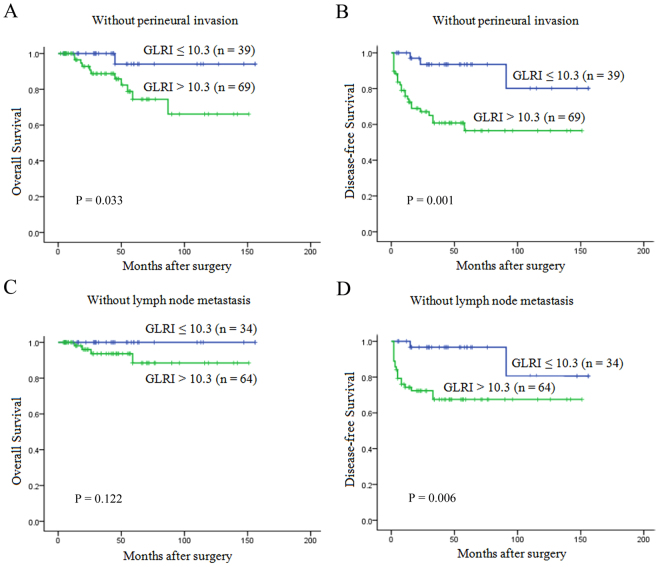
Kaplan-Meier survival curves for the different NF-PNET subgroups. GLRI > 10.3 was significantly correlated with shorter OS and DFS in subgroups without perineural invasion (**A** and **B**). In addition, preoperative GLRI was not a significant prognostic indicator of OS (**C**), while GLRI > 10.3 was a prognostic factor for poor DFS in patients without lymph node metastasis (**D**).

Increasing evidence has suggested that tumor size, stage, perineural invasion and LN involvement were associated with prognosis in patients with PNET, we next investigated the prognostic value of the preoperative GLRI in different subgroups of NF-PNET patients to exclude these factors. The results showed that preoperative GLRI was a prognostic indicator for OS (1-, 3-, 5-year rates: 100%, 100%, 100% vs 96.7%, 88.5%, 74.7%, P = 0.009, respectively) and DFS (100%, 97.1%, 97.1% vs 82.3%, 68.2%, 63.6%, P = 0.003, respectively) in patients with AJCC stage I-II (Fig. 2A,B). Furthermore, in the patients with tumor size > 2 cm, preoperative GLRI > 10.3 also appeared to have notable prognostic value in predicting poorer OS (100%, 100%, 92.9% vs 93.2%, 79.1%, 64.3%, P = 0.012, respectively) and DFS (100%, 91.5%, 91.5% vs 67.2%, 48.9%, 45.1%, P = 0.011, respectively) (Fig. 2C,D), and this prognostic value of OS (100%, 100%, 94.1% vs 96.5%, 88.7%, 74.4%, P = 0.033, respectively) and DFS (100%, 93.5%, 93.5% vs 75.8%, 60.8%, 56.5%, P = 0.001, respectively) also existed in patients without perineural invasion (Fig. 3A,B). In addition, preoperative GLRI was not a significant prognostic indicator of OS (P = 0.122) (Fig. 3C), while GLRI > 10.3 was a prognostic factor for poor DFS in the patients without LN metastasis (100%, 96.7%, 96.7% vs 74.2%, 67.5%, 67.5%, P = 0.006, respectively) (Fig. 3D).

### Comparative performance of GLRI and other predictive models

**Figure 4:**
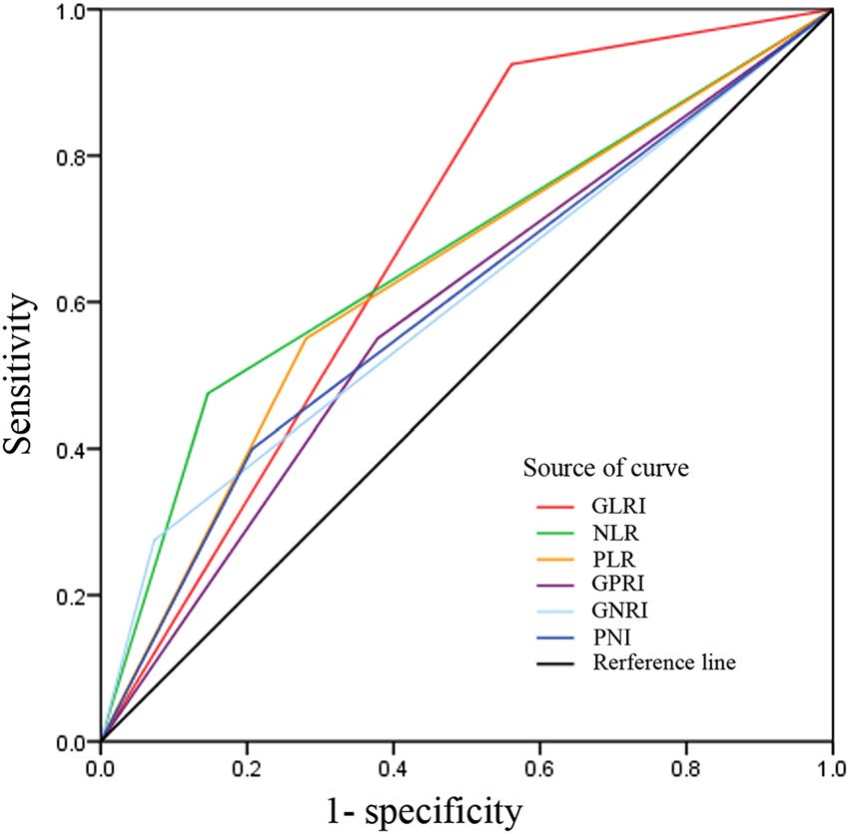
Comparison of the area under the receiver operating characteristic curve (AUC) in different inflammation-based scores. The discriminatory capability of GLRI was superior to that of other inflammation-based scores in OS prediction.

To further evaluate the prognostic value of GLRI, other inflammation-based scores and conventional stratification systems, ROC analysis was performed, and AUC values were compared. The GLRI had a higher AUC value (0.682; P = 0.001) than GNRI, GPRI, PLR, NLR and PNI (Fig. 4). However, the conventional staging systems, including AJCC staging and WHO classification, were superior to GLRI in OS prediction for NF-PNET (Table 4). In addition, the predictive ability of the AJCC staging systems was superior to the WHO classification in our cohort (AUC value: 0.738 vs 0.701).

**Table 4:** Areas under the ROC curve for conventional staging systems and inflammation-based prognostic scores for predicting OS in NF-PNET undergoing radical resection.

Variables	Area under the ROC curve (95% CI)	P
Combined predictive models		
AJCC stage + GLRI	0.814 (0.732–0.896)	**<0.001**
WHO classification + GLRI	0.806 (0.729–0.884)	**<0.001**
AJCC stage + WHO classification	0.831 (0.752–0.909)	**<0.001**
AJCC stage + NLR	0.805 (0.717–0.893)	**<0.001**
WHO classification + NLR	0.783 (0.699–0.868)	**<0.001**
AJCC stage + PLR	0.791 (0.702–0.880)	**<0.001**
WHO classification + PLR	0.755 (0.670–0.841)	**<0.001**
Staging systems		
AJCC stage	0.738 (0.631–0.844)	**<0.001**
WHO classification	0.701 (0.609–0.792)	**<0.001**
Inflammation-based scores		
GLRI (≤10.3/>10.3)	0.682 (0.587–0.777)	**0.001**
GNRI (≤28.1/>28.1)	0.601 (0.489–0.713)	0.071
GPRI (≤0.15/>0.15)	0.586 (0.478–0.694)	0.124
NLR (≤2.6/>2.6)	0.664 (0.556–0.773)	**0.003**
PLR (≤140.88/>140.88)	0.635 (0.527–0.742)	**0.016**
PNI (≤46.3/>46.3)	0.581 (0.472–0.689)	0.149

ROC, receiver operating characteristic; OS, overall survival; NF-PNET, nonfunctional pancreatic neuroendocrine tumor; AJCC, American Joint Committee on Cancer; GLRI, gamma-glutamyltransferase to lymphocyte ratio index; GNRI, gamma-glutamyltransferase to neutrophil ratio index; GPRI, gamma-glutamyltransferase to platelet ratio index; NLR, neutrophil to lymphocyte ratio; PLR, platelet to lymphocyte ratio; PNI, prognostic nutritional index. P-values < 0.05, marked in bold font, indicate statistical significance.

The model integrating the GLRI and the AJCC stage for OS prediction yielded higher AUC values than the AJCC stage alone (0.814 vs 0.738), while the predictive ability of the model integrating the GLRI and the WHO classification was superior to the WHO classification alone (0.806 vs 0.701). In addition, the model including the AJCC stage and WHO classification had the highest AUC values, followed by the model integrating the GLRI and AJCC stage (Table 4).

## Discussion

The present study showed that preoperative GLRI, a novel and easily accessible inflammation-based score derived from serum GGT and lymphocytes,was a predictor of survival in patients with NF-PNET following curative resection. Furthermore, we observed that elevated preoperative GLRI was associated with advanced tumor stages. Finally, we showed that GLRI outperformed other inflammation-based scores in terms of discriminatory capacity. The predictive models incorporating GLRI and conventional stratification systems, including the WHO classification and AJCC stage, showed improved predictive power, compared to stratification systems alone.

Recently, increasing evidence has confirmed that tumor development is associated with inflammation and immunity. Inflammation plays an important role in tumor growth^21–23^. GGT was reported to play a prooxidant role, and the subsequent production of reactive oxygen species (ROS) could promote certain intracellular and extracellular molecular signals^24^. ROS have been reported to promote an epithelial-to-mesenchymal transition via the Snail-E-cadherin pathway and to induce inflammation via the nuclear factor kappa B pathway^25–27^. Therefore, the GGT level has been characterized as a biomarker for oxidative stress and has been shown to be correlated with inflammation in the extracellular tissue microenvironment. It has been found that GGT plays a pivotal role in tumor progression and aggressiveness, and it acts as a significant prognostic biomarker in several cancer entities^9–11^.

In addition, lymphocytes, a marker of patient immune status, play a central role in the systemic inflammatory response. A number of studies have demonstrated a relationship between the lymphocyte count or neutrophil-lymphocyte ratio and the prognosis of cancer patients^28–30^.

This association can be explained by CD4+ T lymphocyte cells acting as a sensor in detecting precancerous cells and then regulating their eradication^31^. The loss of CD4+ T lymphocytes accompanied by the impaired activation of CD8+ T lymphocyte cells can cause the insufficient secretion of cytotoxin performing anti-carcinogenic functions in neoplastic microenvironments^32^. Therefore, GLRI, a novel inflammatory marker that reflects the status of oxidative stress and immune function, could be a potential predictive marker.

In our study, we first identified the cut-off value of preoperative GLRI according to the ROC curve, and 10.3 appeared to be the optimal cut-off value for GLRI with maximum joint sensitivity and specificity. Notably, concerning the correlations between GLRI and clinical characteristics, we found that an elevated GLRI was positively correlated with gender, presence of symptoms, AKT, T-stage, perineural invasion, and AJCC stage. Moreover, patients with elevated GLRI were more inclined to have a higher rate of distant metastasis. All of these data showed that GLRI could reflect the tumor burden and tumor progression. With further analysis, we found that elevated GLRI was identified as an independent risk factor for OS and DFS in this cohort. The 1-, 3- and 5-year OS rates and DFS rates of patients with high levels of GLRI were markedly lower than those in the low level group. For the subgroup of patients with stage I/II, we also found that a preoperative GLRI > 10.3 was predictive of significantly worse survival. Thus, the preoperative GLRI might be able to predict poor prognosis in patients with early NF-PNET. In addition, in the subgroup with tumor size >2 cm or in patients without perineural invasion, preoperative GLRI > 10.3 also showed prognostic value in predicting poorer OS and DFS. All of these data provided further evidence that preoperative GLRI could act as a potential prognostic marker to predict survival in NF-PNET patients undergoing curative resection, in accordance with our above hypothesis.

TNM stage, grade or markers of systemic inflammation, such as the NLR and PLR, have been emerged as prognostic indicators in PNET^1,2,30^, similar to our study. To the best of our knowledge, the existing stratification systems and predictive models for PNET, including the above mentioned TNM staging system, WHO classification and two nomograms^33,34^, lacked indicators of systemic inflammation, which could offer additional information in prognostic evaluation. Han X *et al*. reported that an effective nomogram including the chromogranin A level, liver metastases tumor burden, and Ki-67, predicted overall survival in well-moderate NF-PNETs with liver metastases, and the nomogram showed fitting calibration with a C-index of 0.87 (95% confidence interval, 0.82–0.92)^33^. Furthermore, the research from Ellison TA *et al*. had confirmed that a simple prognostic nomogram, including continuous Ki-67 labelling, sex, and age at surgery (≤63, >63) could be used to predict survival for NF-PNETs with a Harrel’s c-index value of 0.74^34^. Herein, we incorporated GLRI, an inflammation-based biomarker, into AJCC staging and WHO classification and showed that the predictive abilities of models integrating the GLRI and the stratification systems for OS was superior to the stratification systems alone. The results were supportive of the integration of GLRI into conventional stratification systems for improved discriminative ability.

Our study had several limitations that must be considered. First, the present study was retrospective in nature and a single-centre experience. Second, only patients who underwent curative resection were included in the study. Furthermore, due to the limited number of patients, external validation was not performed. Therefore, future studies should be performed to evaluate the prognostic significance of GLRI in patients with advanced clinical stages and different treatment modalities.

In conclusion, as a novel and easily accessible inflammation-based biomarker, preoperative GLRI was an independent predictor of OS and DFS for NF-PNET patients undergoing curative resection. Furthermore, we confirmed that prognostic models incorporating GLRI into the TNM staging system or WHO classification provided improved predictive accuracy, compared with the stratification systems alone. Therefore, we recommend that surgeons devise the treatment plans considering not only TNM stage but also these prognosis related serum biomarkers. Only in this manner can we acquire better personalised therapy for patients with NF-PNET.

## Materials and Methods

### Study population

Patients who underwent curative resection for NF-PNET from November 2003 to August 2016 at the First Affiliated Hospital, Zhejiang University School of Medicine, were retrospectively reviewed. The diagnosis of PNET was made based on standard histologic criteria. Patients were excluded if they: (1) showed clinical evidence of infection or evidence of hyperpyrexia at the time of diagnosis; (2) were treated for recurrent disease; (3) received preoperative radiochemotherapy prior to surgery; (4) underwent an R1 or R2 resection; (5) had a history of cancer of any type; and (6) did not consent to the use of their medical records for research purposes. We included in the study only those patients who had survived for at least 60 days after surgery in the study to exclude perioperative mortality-related bias. The study was approved by the Ethics Committee of the First Affiliated Hospital of Zhejiang University School of Medicine and was conducted in accordance with the Declaration of Helsinki. Written informed consent was obtained from all of the participants before the commencement of the study. All of the methods and research activities were performed in accordance with the guidelines and regulations.

Laboratory tests included routine blood testing, albumin, GGT and AKT, which were routinely performed within 7 days before the surgical resection and 7 days after the operation. The TNM stage of each PNET was determined based on the American Joint Committee on Cancer (AJCC) TNM classification, while the grade of each PNET was determined according to the 2010 WHO classification of NETs of the GEP system. Six inflammatory factors, including GLRI, GPRI, GNRI, NLR, PLR and PNI, were included in this analysis. The definitions of the six inflammatory factors are as follows: NLR = neutrophil count/lymphocyte count; PLR = platelet count/lymphocyte count; PNI = serum albumin levels (g/dl) × 10 + total lymphocyte count (per mm^3^) × 0.005; GLRI = (GGT value/lymphocyte count) × 10^9^/U; GPRI = (GGT value/platelet count) × 10^9^/U; GNRI = (GGT value/neutrophil count) × 10^9^/U.

### Follow-up

Patient follow-up was performed by reviewing hospital records or contacting patient family members. Overall survival (OS) was defined as the time span extending from the date of the initial diagnosis until the date of death from any cause or the date of last known contact. Disease-free survival (DFS) was defined as the time extending from the date of surgery to the date of PNET recurrence. Patients who did not have evidence of local recurrence or metastasis at the last follow-up and patients who died of diseases unrelated to PNET were censored from the analysis of DFS. Our department follows up with patients every 6 months for the first 5 years after surgery and then yearly thereafter. The following postoperative follow-up data were collected for each patient: clinical symptoms and signs; laboratory test results; and radiological examination results.

### Statistical analysis

All of the statistical analyses were performed using SPSS software, version 16.0 for Windows (SPSS, Chicago, IL, USA). Area under the curve (AUC) values, obtained from receiver operating characteristic (ROC) curve analysis, were used to compare the predictive efficacies of GLRI and the afore mentioned inflammatory factors. Differences between groups were analysed using Pearson’s chi-square test, Fisher’s exact test or the Mann-Whitney U test as appropriate. The Kaplan-Meier method and the log-rank test were used to calculate OS and DFS, respectively. Prognostic analysis was performed using univariate and multivariate Cox regression models. A P-value < 0.05 was considered statistically significant. All of the data generated or analysed during this study are included with this published article.

## References

[1] Klimstra DS, Modlin IR, Coppola D, Lloyd RV, Suster S (2010). The pathologic classification of neuroendocrine tumors: a review of nomenclature, grading, and staging systems. Pancreas.

[2] Fischer L (2008). Clinical outcome and long-term survival in 118 consecutive patients with neuroendocrine tumors of the pancreas. Br J Surg.

[3] Bilimoria KY (2008). Prognostic score predicting survival after resection of pancreatic neuroendocrine tumors: analysis of 3851 patients. Ann Surg.

[4] Yang M (2014). Surgical treatment and clinical outcome of nonfunctional pancreatic neuroendocrine tumors: a 14-year experience from one single center. Medicine (Baltimore).

[5] Strosberg JR (2012). Relapse-free survival in patients with nonmetastatic, surgically resected pancreatic neuroendocrine tumors: an analysis of the AJCC and ENETS staging classifications. Ann Surg.

[6] Strosberg JR (2011). Prognostic validity of a novel American Joint Committee on Cancer Staging Classification for pancreatic neuroendocrine tumors. J Clin Oncol.

[7] Bettini R (2008). Prognostic factors at diagnosis and value of WHO classification in a mono-institutional series of 180 non-functioning pancreatic endocrine tumors. Ann Oncol.

[8] Corti A, Franzini M, Paolicchi A, Pompella A (2010). Gamma-glutamyltransferase of cancer cells at the crossroads of tumor progression, drug resistance and drug targeting. Anticancer Res.

[9] Everhart JE, Wright EC (2013). Association of γ-glutamyl transferase (GGT) activity with treatment and clinical outcomes in chronic hepatitis C (HCV). Hepatology.

[10] Engelken FJ, Bettschart V, Rahman MQ, Parks RW, Garden OJ (2003). Prognostic factors in the palliation of pancreatic cancer. Eur J Surg Oncol.

[11] Yin X (2013). Elevation of serum γ-glutamyltransferase as a predictor of aggressive tumor behaviors and unfavorable prognosis in patients with intrahepatic cholangiocarcinoma: analysis of a large monocenter study. Eur J Gastroenterol Hepatol.

[12] Ma H (2014). γ-Glutamyltranspeptidase is a prognostic marker of survival and recurrence in radiofrequency-ablation treatment of hepatocellular carcinoma. Ann Surg Oncol.

[13] Diergaarde B (2010). Pooling-based genome-wide association study implicates gamma-glutamyltransferase 1 (GGT1) gene in pancreatic carcinogenesis. Pancreatology.

[14] Strasak AM (2008). Prospective study of the association of gamma-glutamyltransferase with cancer incidence in women. Int J Cancer.

[15] Clark EJ (2007). Preoperative lymphocyte count as a prognostic factor in resected pancreatic ductal adenocarcinoma. HPB (Oxford).

[16] Xiao Y (2016). Neutrophil and lymphocyte counts at diagnosis are associated with overall survival of pancreatic cancer: A retrospective cohort study. Medicine (Baltimore).

[17] Katz SC (2010). T cell infiltrate and outcome following resection of intermediate-grade primary neuroendocrine tumours and liver metastases. HPB (Oxford).

[18] Katz SC (2009). T cell infiltrate predicts long term survival following resection of colorectal cancer liver metastases. Ann Surg Oncol.

[19] Boyd A, Bottero J, Lacombe K (2016). The γ-glutamyl transpeptidase-to-platelet ratio as a predictor of liver fibrosis in patients co-infected with HBV and HIV. Gut.

[20] Wang WL (2016). Preoperative γ-glutamyl transpeptidase to platelet ratio (GPR) is an independent prognostic factor for HBV-related hepatocellular carcinoma after curative hepatic resection. Medicine (Baltimore).

[21] Shimizu T, Marusawa H, Endo Y, Chiba T (2012). Inflammation-mediated genomic instability: roles of activation-induced cytidine deaminase in carcinogenesis. Cancer Sci.

[22] Grivennikov SI, Greten FR, Karin M (2010). Immunity, inflammation, and cancer. Cell.

[23] Coussens LM, Werb Z (2002). Inflammation and cancer. Nature.

[24] Stark AA (1994). Localization of oxidative damage by a glutathione-gamma-glutamyl transpeptidase systemin preneoplastic lesions in sections of livers from carcinogen-treated rats. Carcinogenesis.

[25] Larue L, Bellacosa A (2005). Epithelial-mesenchymal transition in development and cancer: role ofphosphatidylinositol3’ kinase/AKT pathways. Oncogene.

[26] Javed S, Mejías-Luque R, Kalali B, Bolz C, Gerhard M (2013). Helicobacter bilis gamma-glutamyltranspeptidase enhances inflammatory stress response via oxidative stress in colon epithelial cells. PLoS One.

[27] Wu Y, Zhou BP (2010). TNF-alpha/NF-kappaB/Snail pathway in cancer cell migration and invasion. Br J Cancer.

[28] Zhang J (2013). Preoperative lymphocyte count is a favorable prognostic factor of disease-free survival in non-small-cell lung cancer. Med Oncol.

[29] Milne K (2012). Absolute lymphocyte count is associated with survival in ovarian cancer independent of tumor-infiltrating lymphocytes. J Transl Med.

[30] Salman T (2016). Prognostic Value of the Pretreatment Neutrophil-to-Lymphocyte Ratio and Platelet-to-Lymphocyte Ratio for Patients with Neuroendocrine Tumors: An Izmir Oncology Group Study. Chemotherapy.

[31] Komura T (2015). Inflammatory features of pancreatic cancer highlighted by monocytes/macrophages and CD4+ T cells with clinical impact. Cancer Sci.

[32] Zhang S (2013). Mesothelin virus-like particle immunization controls pancreatic cancer growth through CD8+ T cell induction and reduction in the frequency of CD4+ foxp3+ ICOS- regulatory T cells. PLoS One.

[33] Han X (2015). The value of serum chromogranin A as a predictor of tumor burden, therapeutic response, and nomogram-based survival in well-moderate nonfunctional pancreatic neuroendocrine tumors with liver metastases. Eur J Gastroenterol Hepatol.

[34] Ellison TA (2014). A single institution’s 26-year experience with nonfunctional pancreatic neuroendocrine tumors: a validation of current staging systems and a new prognostic nomogram. Ann Surg.

